# Clinical utility of molecular profiling in rare lung neuroendocrine neoplasms

**DOI:** 10.3389/fonc.2026.1840985

**Published:** 2026-08-10

**Authors:** Daphne Leunissen, Laura Moonen, Tijmen van Weert, Frank Heijboer, Lisa M. Hillen, Jan Von der Thüsen, Ernst-Jan Speel, Anne-Marie Dingemans, Jules Louis Derks

**Affiliations:** 1 Department of Pathology, GROW Research Institute for Oncology and Reproduction, Maastricht University Medical Centre, Maastricht, Netherlands; 2 Department of Respiratory Medicine, Erasmus MC Cancer Institute, University Medical Center, Rotterdam, Netherlands; 3 Department of Pathology and Clinical Bioinformatics, Erasmus University Medical Center, Rotterdam, Netherlands

**Keywords:** biomarkers, clinical management, LCNEC, lung neuroendocrine neoplasms, molecular profiling, pulmonary carcinoids

## Abstract

Rare lung neuroendocrine neoplasms (LNENs), including pulmonary carcinoids and large-cell neuroendocrine carcinomas (LCNEC), are heterogeneous tumors. Current World Health Organization (WHO) classification primarily relies on morphological features, leading to important inter-observer variability and sub-optimal prognostic accuracy. This review highlights the clinical utility of molecular profiling to overcome these diagnostic and prognostic challenges. Recent multi-omics studies have demonstrated that pulmonary carcinoids are not a uniform entity but comprise distinct molecular subgroups (A1, A2, B, and supra-carcinoids) with unique clinical and genomic features. Furthermore, the recently described atypical SCLC adds another layer of heterogeneity to this spectrum. For clinical practice, a biomarker panel consisting of OTP, CD44, and Ki-67 can improve the prediction of disease recurrence in pulmonary carcinoids, enabling personalized follow-up strategies. Furthermore, subgroup-specific markers, such as OTP, ASCL1, and HNF1A, may facilitate clinical implementation of molecular profiles. Within LCNEC molecular profiles are heterogenous, generally defining two major subgroups (SCLC-like and NSCLC-like). Emerging therapies targeting *DLL3* and *SEZ6* show potential relevance for clinical screening of these targets. Integration of subgroup-specific molecular markers into routine diagnostics is essential. This approach may allow clinicians to refine risk stratification, prevent unnecessary long-term surveillance, and develop personalized treatment approaches for patients with pulmonary NENs.

## Introduction

Lung neuroendocrine neoplasms (LNENs) are heterogeneous tumors of which the majority originates from cells within the neuroendocrine (NE) system. NENs can be divided into well-differentiated neuroendocrine tumors (NETs) and poorly differentiated neuroendocrine carcinomas (NECs). This review focuses on rare lung NETs (LNET), also known as pulmonary carcinoids, and large-cell neuroendocrine carcinoma (LCNEC).

**Figure 1:**
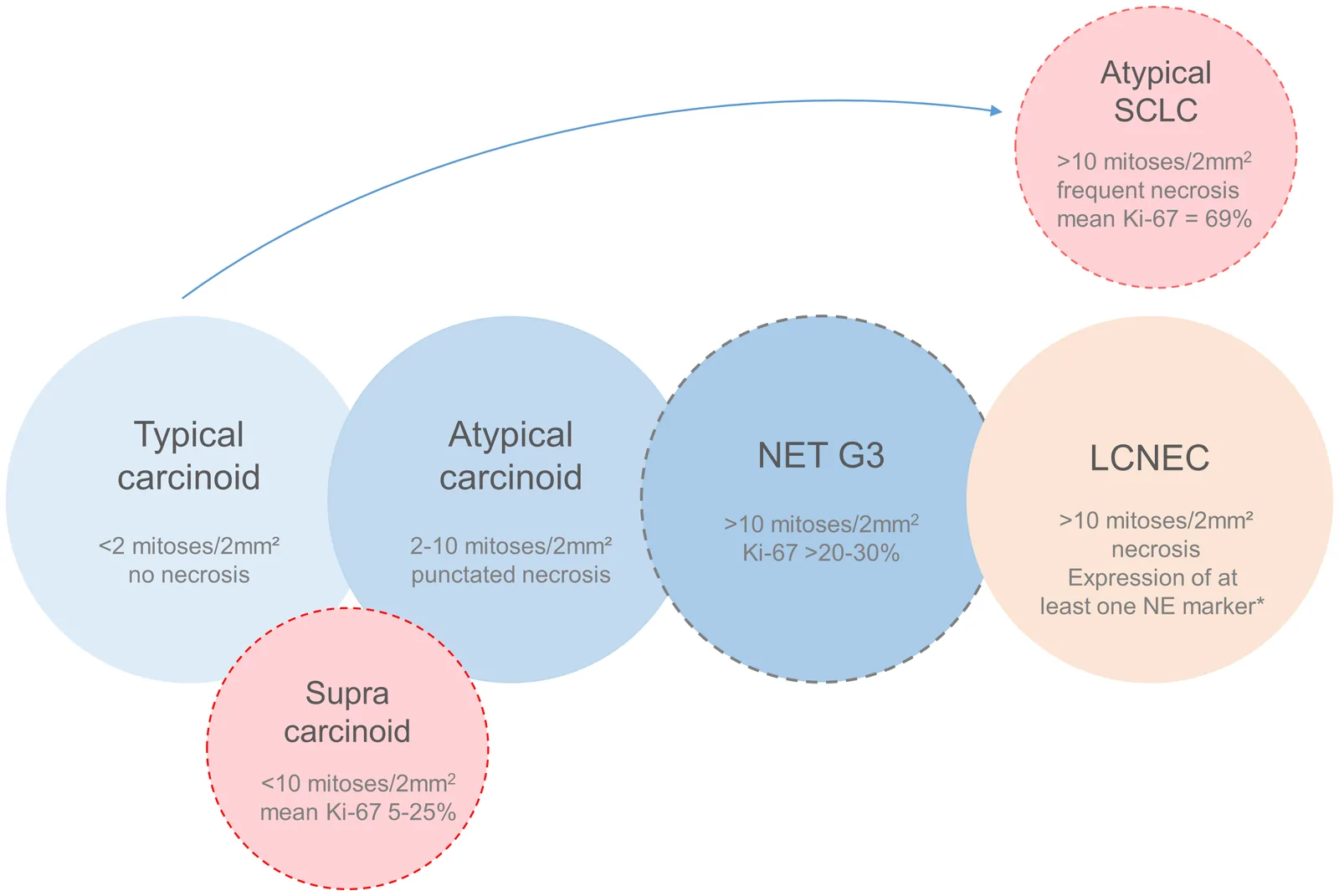
Schematic overview of the diagnostic spectrum of WHO guidelines of rare lung NENs comprising of pulmonary carcinoids (NET G1 and NET G2), NET G3 tumors (provisional entity), LCNEC and the two new entities; supra-carcinoids and atypical SCLC. *NE markers: chromogranin A, synaptophysin, INSM1, and CD56 (46). Entities are arranged horizontally according to their morphological characteristics. Supra-carcinoids show morphological overlap with lower-grade carcinoids. In contrast, atypical SCLC (aSCLC) is morphologically distinct and more closely resembles conventional SCLC. Therefore, aSCLC is shown separately but remains connected to carcinoids by an arrow, reflecting their proposed molecular relationship. Dashed outlines indicate entities that are not currently recognized in the WHO classification. Red shading denotes biologically more aggressive entities.

Using the World Health Organization (WHO) classification, carcinoids can be classified into low-grade typical carcinoid and intermediate-grade atypical carcinoid based on mitotic count and presence of necrosis (**Figure 1**). Typical carcinoids are less aggressive and classified by the absence of necrosis and <2 mitosis/2 mm^2^, while atypical carcinoids show ≥2–10 mitoses/2 mm^2^ and necrosis (1). Although rare, the incidence of typical and atypical carcinoids continues to increase, comprising 1-2% and 0.2% of all lung cancers, respectively (2). As most LNETs can be treated curatively, the clinical challenge is to tailor follow-up strategies to the individual patient’s tumor characteristics, ensuring that patients receive surveillance tailored to the biological behavior of the tumor. The 5-year overall survival for patients with typical carcinoids ranges from 82-100%, whereas patients with atypical carcinoid demonstrate worse outcomes, with survival rates of 50% (3). To confirm the diagnosis, the WHO recommends immunohistochemical (IHC) staining of synaptophysin, chromogranin A, and CD56 (1). Although Ki-67 is incorporated into grading systems for several non-pulmonary NENs (e.g., head & neck NETs and pancreatic and gastrointestinal (GEP) NETs) (4), this is currently not yet the case for pulmonary carcinoids. Recently, several studies have highlighted the potential clinical relevance of Ki-67 as a prognostic marker in pulmonary carcinoids, and its incorporation into future WHO guidelines is recently suggested (5).

**Figure 2:**
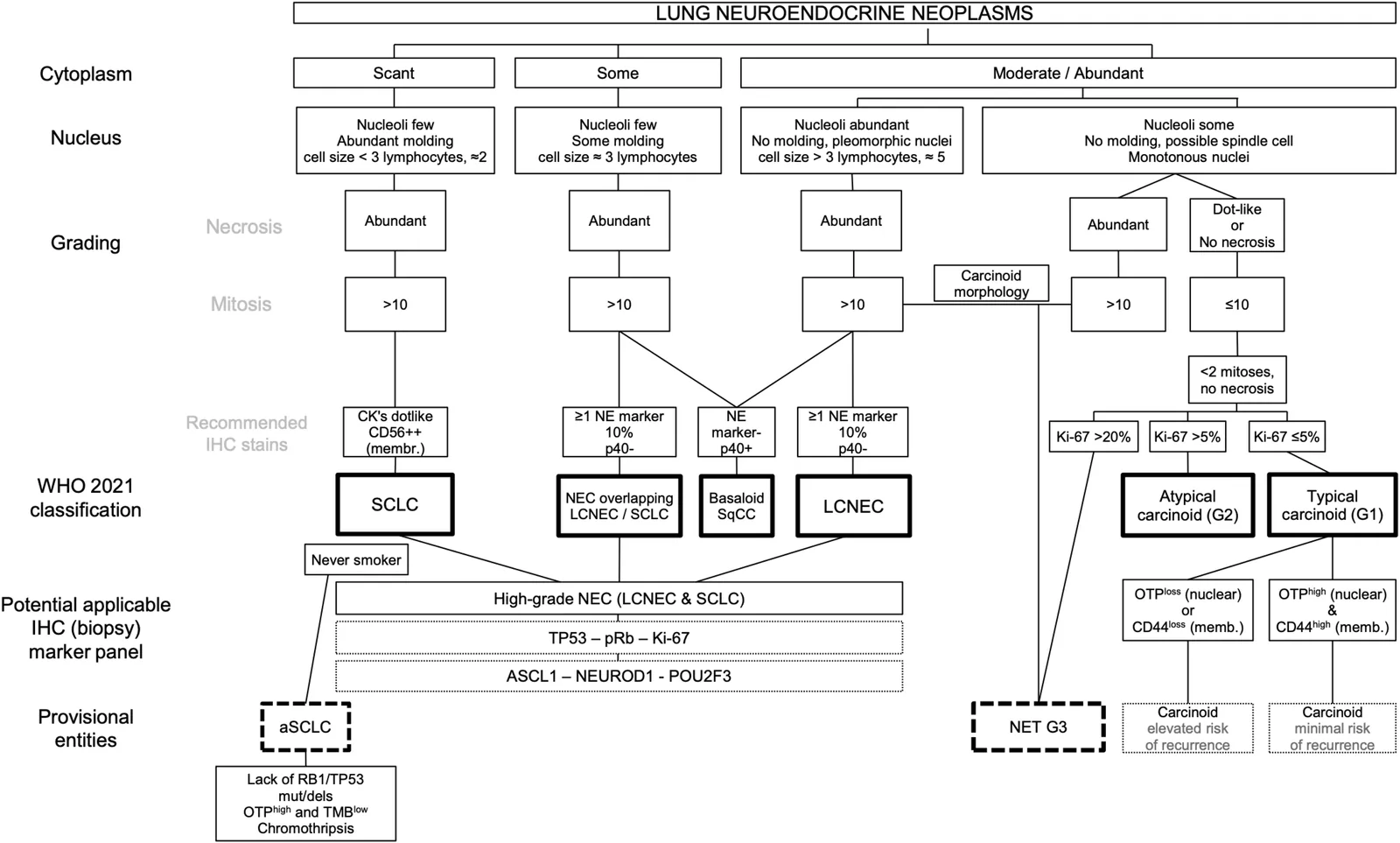
Diagnostic workflow for the classification of pulmonary NENs based on the WHO 2021 criteria and additional molecular markers. CK, cytokeratines; IHC, immunohistochemistry; LCNEC, large-cell neuroendocrine carcinoma; membr, membranous; NE, neuroendocrine; NEC, neuroendocrine; NEN, neuroendocrine neoplasm; SqCC, squamous cell carcinoma; SCLC, small cell lung cancer; aSCLC, atypical SCLC; mut, mutations; dels, deletions.

Unlike non-pulmonary NENs, which follow a three-tiered grading system (G1–G3), LNETs are still categorized as grade 1 (typical carcinoid) and grade 2 (atypical carcinoid), although a subset of tumors with features consistent with grade 3 NET have been proposed (6–10). These tumors are characterized by carcinoid morphology but are clinically more aggressive than atypical carcinoids, with >10 mitoses/2 mm^2^ and/or increased Ki-67 index of >20%, resulting in a poorer prognosis (11). In addition to G3-NET, another emerging entity is the grade 1–3 NET with a molecular profile similar to LCNEC defined as ‘supra-carcinoid’ (12, 13). Lastly, an additional layer of heterogeneity with the origin of a carcinoid has been suggested through the identification of “atypical small cell lung cancer (SCLC)” (aSCLC) (14). These tumors have the morphology of SCLC, but show a histogenetic relationship with carcinoids, sharing some genomic alterations and marker expression profiles, and a more aggressive course of disease. aSCLC are characterized by a mitotic count of >10 mitoses/2 mm^2^, extensive necrosis, a high Ki-67 ranging from 70% to 90%, and expression of multiple NE markers and often that of OTP (14). Hence, recent studies have provided more insight into unique aggressive biological subgroups of carcinoids increasing our understanding. **Figure 2** illustrates the diagnostic workflow for the classification of the aforementioned tumor types according to the WHO criteria with integration of the newly proposed entities.

Large cell neuroendocrine carcinoma (LCNEC) is a clinically aggressive tumor associated with a poorer prognosis compared to pulmonary carcinoids and comparable to SCLC in terms of clinical behavior and outcomes (15). The diagnosis of LCNEC is based on a high mitotic count (>10 mitoses/2 mm^2^), necrosis, and expression of at least one NE marker, e.g., synaptophysin, chromogranin A, or CD56 (1). LCNECs are rare tumors, accounting for approximately 1%–3% of all lung cancers; however, their incidence appears to be increasing, and they frequently present with metastatic disease at the time of diagnosis (16). Treatment typically consists of surgical resection combined with adjuvant chemotherapy for local disease, while limited effective palliative systemic treatment options are available in the metastatic setting (>50% of *de novo* presentations). Patients with LCNEC have a median overall survival between 7 and 12 months (17–19), and they are often challenging to diagnose (15). Within LCNEC, the main clinical challenge is to correctly diagnose it and find the most appropriate systemic therapy, adapted to the biological behavior of the tumor.

In general, the diagnosis of LNENs can be challenging and is subject to considerable inter-observer variability (1, 20). Carcinoid classification primarily relies on morphological assessment of growth patterns, mitotic count, and the presence/absence of necrosis, whereas LCNEC diagnosis requires a combination of morphology and IHC analysis (1, 15). Overlap within this spectrum is common, and the biology of the disease does not reflect the strict cut-offs provided by the current classification. **Figure 1** illustrates the overlap between the different LNENs, including several recently identified molecular entities.

The management of patients with carcinoid and LCNEC is challenging due to clinical and molecular heterogeneity (21). Recent studies have expanded our understanding of the molecular heterogeneity and can aid in diagnostic and prognostic assessment. Importantly, the clinical course of patients with tumors of the same grade or stage may differ, underscoring the need for biomarkers to complement patient management strategies (21). This review summarizes the molecular landscape of rare LNENs but not SCLC, evaluates current evidence, and discusses future directions for clinical management based on recent molecular insights.

## Pulmonary carcinoids

### Molecular subgroups and potential biomarkers

**Figure 3:**
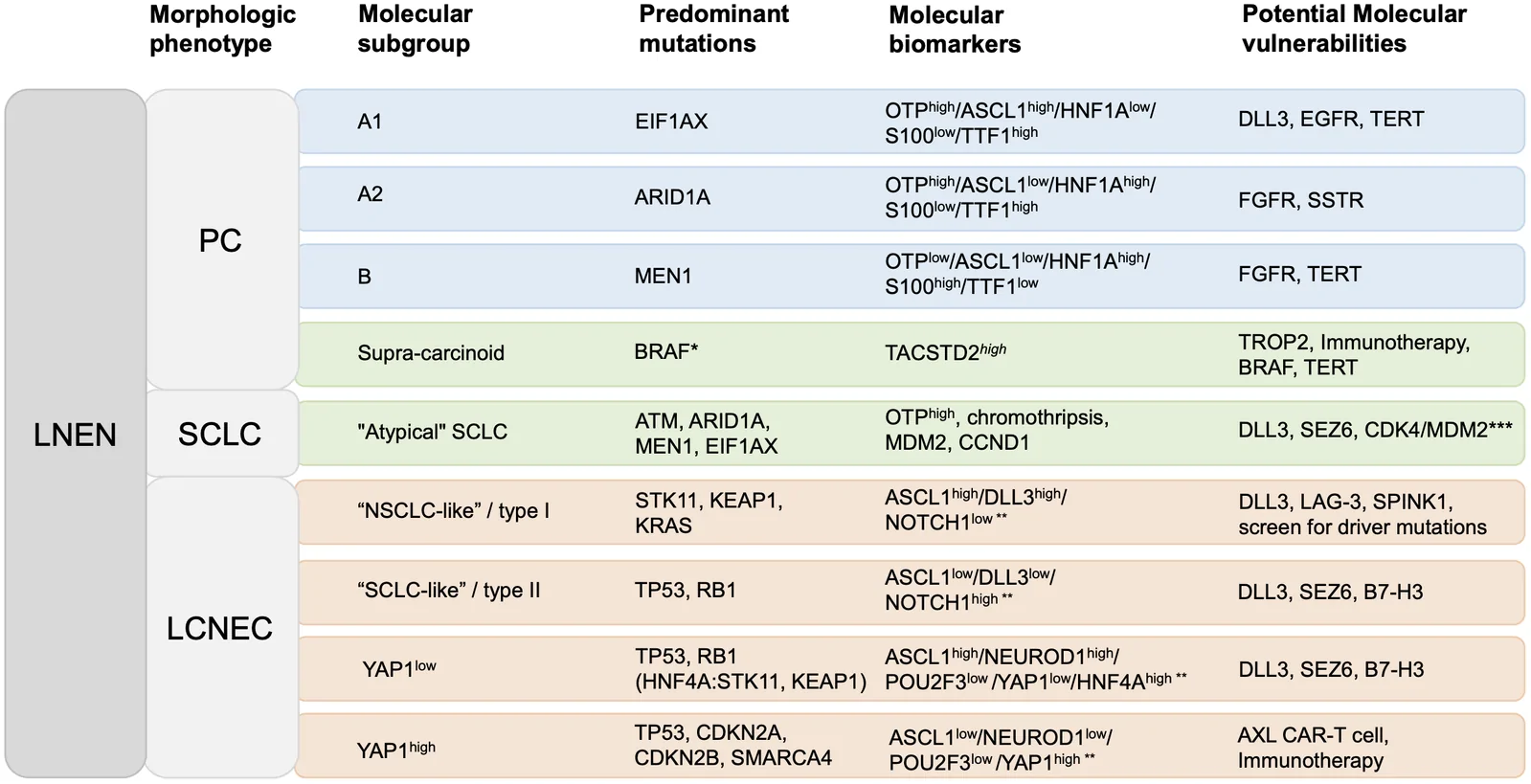
Schematic overview of molecular markers and potential molecular vulnerabilities in rare lung NENs. *Reported in only a small number of cases (n=2/14) (12, 46) and requires further validation. **Findings are not consistent with those reported by Nassar et al. ***agents targeting amplification and overexpression of CDK4 and MDM2 (14). LNEN, Lung Neuroendocrine Neoplasms; PC, Pulmonary Carcinoid; SCLC, Small Cell Lung Cancer; LCNEC, Large Cell Neuroendocrine Carcinoma.

**Figure 4:**
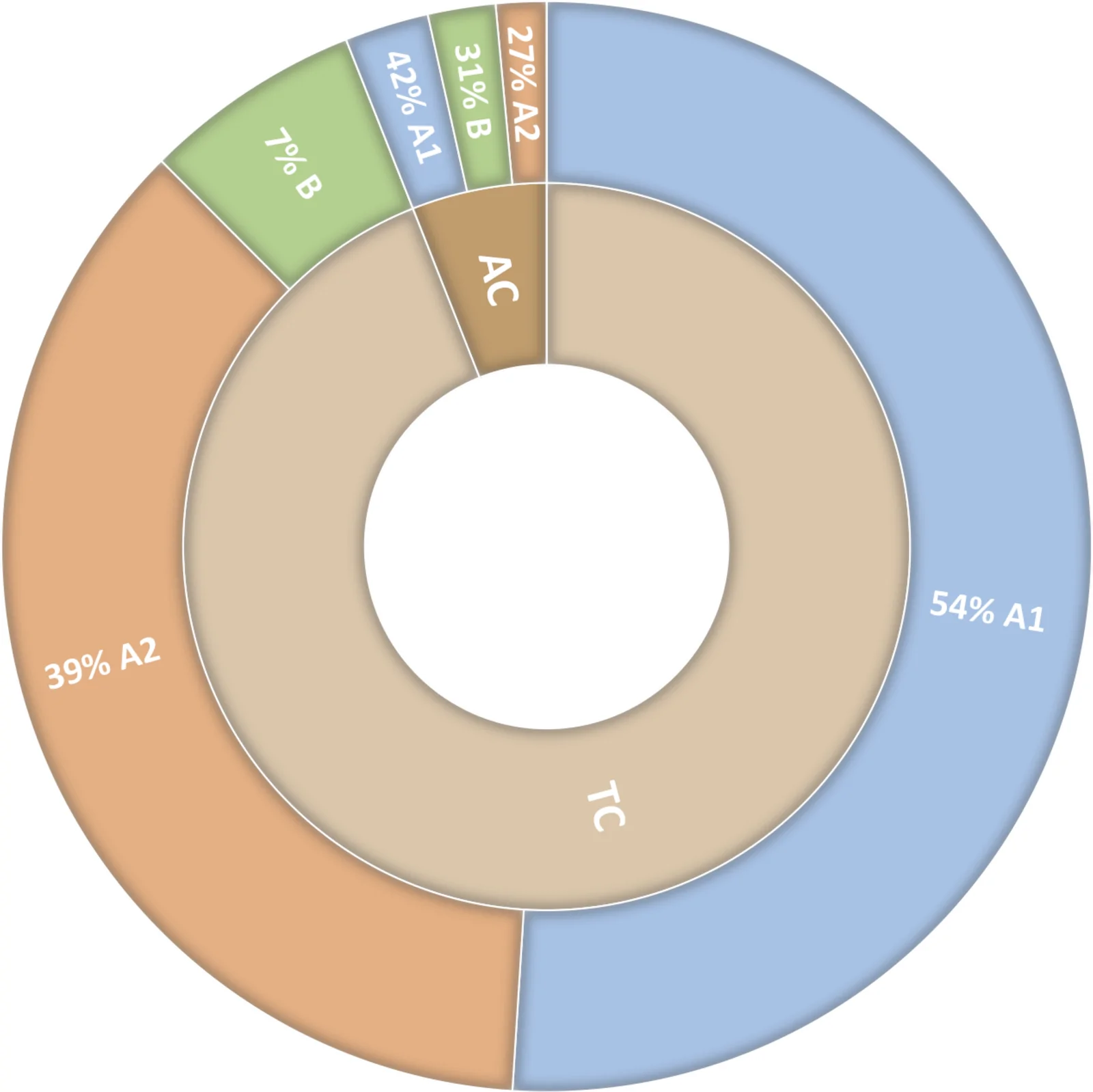
Sunburst diagram illustrating the relationship between the current WHO entities and the molecular subgroups of pulmonary carcinoids, adapted from Leunissen et al.

Pulmonary carcinoids have a low tumor mutational burden (0.4 mutations/Mb) (22). One of the most frequently mutated pathways is the chromatin-remodeling pathway, which regulates gene expression through chromatin structure modification. Mutated genes identified in this pathway include *MEN1*, *ARID1A*, and *EIF1AX* (**Figure 3**) (22). *MEN1* mutations are the most frequent alterations occurring in 13% of pulmonary carcinoids and have been associated with a poorer prognosis (20). Consistent with these findings, a study by Laddha et al. identified *MEN1* and *ARID1A* as the most frequently mutated genes, and additionally reported mutations in methyl transferase genes and genes involved in chromatin-remodeling, i.e., *KMT2A*, *KMT2C*, KMT2D, and *SMARCA4* (23). Similarly, Alcala et al. described mutations in histone modifiers and components of the SWI/SNF complex, as well as in *ATM*, *PSIP1*, and *ROBO1*, and small insertions/deletions affecting *ARID2*, *DOT1L* and *ROBO1* (12). Actionable mutations are uncommon in LNET, occurring in only 1.2% of cases (24). Despite their biological relevance, the most common alterations currently do not represent clinically relevant targets. Therefore, both Laddha et al. and Alcala et al. performed integrated multi-omics analyses, providing additional insights into the molecular landscape of pulmonary carcinoids. These studies independently identified three unique molecular subgroups, referred to as A1 (LC1), A2 (LC3), and B (LC2). Patients with A1 tumors were enriched for females, older age, and peripheral tumors, whereas patients with A2 carcinoid were enriched for younger age and endobronchial (or central) tumors. Patients with subgroup B carcinoid were enriched for males, with the highest rates of recurrence and atypical carcinoid classification (25). **Figure 4** demonstrates the relationship between the WHO categories and the molecular subgroups. Hence, their identification may help to further understand the clinical heterogeneity observed in LNETs.

**Figure 5:**
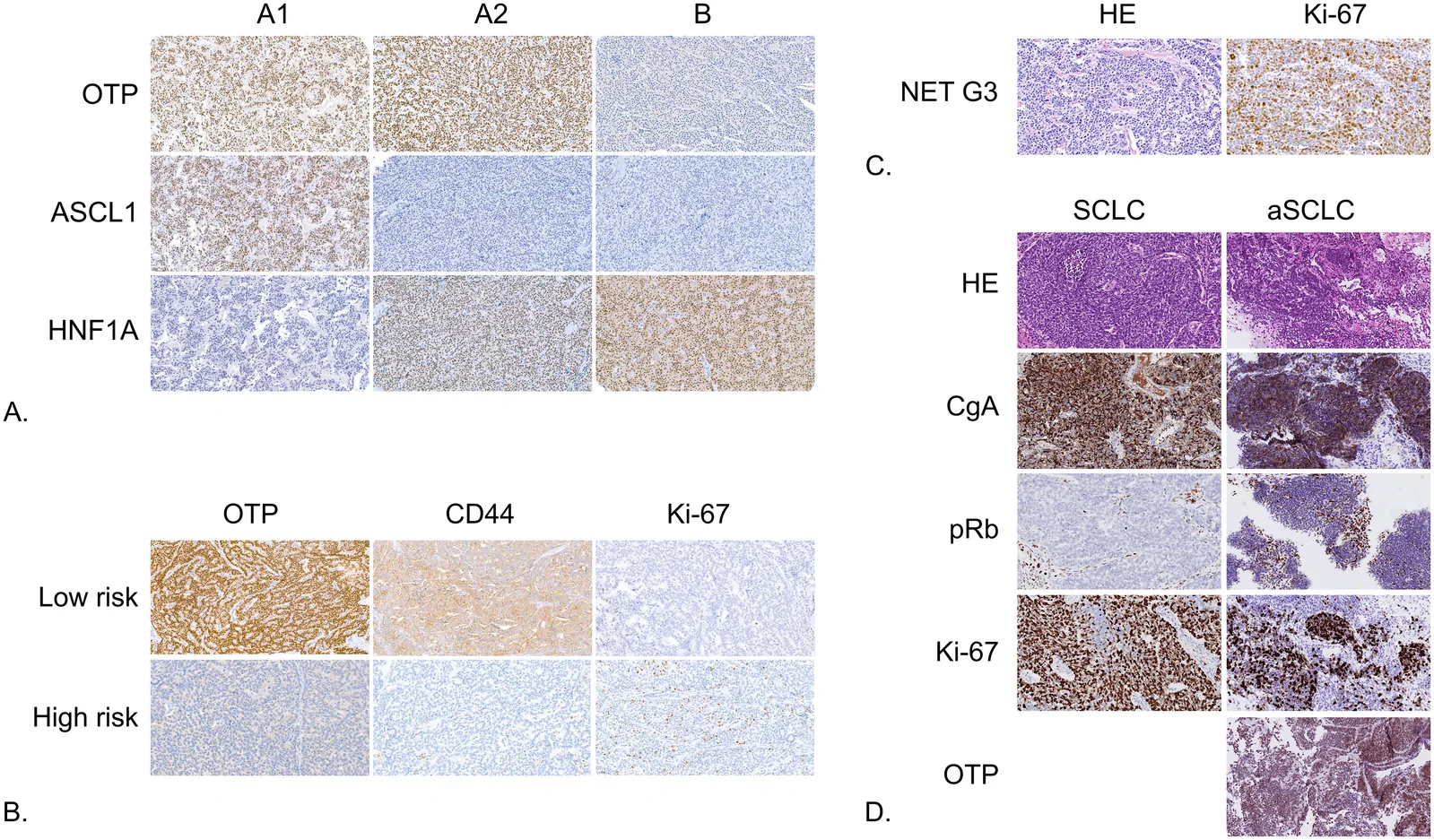
Representative immunohistochemical (IHC) staining patterns. **(A)** Representative expression of OTP, ASCL1, and HNF1A in distinct molecular subgroups of pulmonary carcinoids (magnification ×20) (25) **(B)** Low-risk and high-risk carcinoids based on the OTP/CD44/Ki-67 marker panel (magnification ×20). **(C)** Hematoxylin and eosin (H&E) and Ki-67 staining of a NET G3 case (magnification ×20). **(D)** Representative staining patterns in conventional SCLC and atypical SCLC (aSCLC) (magnification 20x). HE, hematoxylin and eosin; CgA, chromogranin A; pRb, retinoblastoma protein.

Several molecular markers may facilitate clinical implementation. Alcala et al. reported three highly expressed genes, i.e., *OTP*, *ASCL1*, and thyroid transcription factor 1 (*TTF1*), and other genes, such as delta-like canonical Notch ligand 3 (*DLL3*) and angiopoietin-like 3 (*ANGPTL3*). Laddha et al. identified the transcription factors *HNF1A*, *FOXA3*, and *ASCL1* and the calcium-binding protein *S100* to separate molecular subgroups. Recently, IHC staining for orthopedia homeobox protein (OTP), ASCL1, and HNF1A have been described to discriminate molecular subgroups at the protein level, facilitating their implementation in routine clinical practice (25). OTP has been associated with neuroendocrine differentiation and favorable clinical outcome, which is reflected by its high expression in A1 and A2 tumors. The transcription factor ASCL1 is a key regulator of neuroendocrine differentiation and linked to more proliferative tumor types, and predominantly expressed in A1 tumors. HNF1A is involved in metabolic regulation and predominantly expressed in A2 and B tumors (**Figure 5A**) (25). Using an IHC panel of these three markers, up to 88% of pulmonary carcinoids could be classified. In the remaining 12% of cases, the addition of TTF1 and S100 provided further basis for subgrouping. TTF1 is a crucial protein, mainly involved in the development and differentiation of the thyroid, lung and central nervous system (26). S100 is a calcium binding protein involved in tumorigenesis and poor prognosis (23, 27). Other groups have confirmed the identification of molecular groups (21, 28). Also, the relevance of OTP-ASCL1 staining correlating its expression with prognosis has been shown in atypical carcinoids (29). Furthermore, Ura et al. utilized a transcription factor-based classification, defined by OTP and ASCL1 expression, again confirming unique clinical features such as sex, tumor location and hormone expression status (30).

Overall, these findings demonstrate that pulmonary carcinoids comprise more biologically distinct entities than the WHO-defined typical and atypical carcinoids, which can be further subdivided based on their molecular profiles. Therefore, the integration of subgroup-specific biomarkers into routine diagnostics may inform our understanding of clinical heterogeneity and enable a more personalized patient management in the future.

### Clinical utility

Morphology-based diagnostic guidelines for LNENs were introduced in the early 1990s for LCNEC and earlier for pulmonary carcinoids. These have remained largely unchanged since the 1999 WHO classification (31, 32). However, these criteria have important limitations that can lead to clinical dilemmas. First, the current WHO morphologic criteria are associated with considerable interobserver variability. Studies have reported agreement ranging from moderate (κ = 0.60–0.76; agreement among 3 of 5 pathologists) to minimal (κ = 0.32; agreement among all 5 pathologists) when applying the WHO guidelines, which may result in misclassification between typical and atypical carcinoids (20, 33, 34). This is clinically relevant, as follow-up protocols are determined by this classification (1). Second, these criteria can generally be applied only to postoperative resection specimens, which limits the accuracy of preoperative diagnosis and treatment decision-making. Moonen et al. evaluated the reliability of preoperative biopsy specimens for carcinoid classification and reported a discordance of 57% between the preoperative biopsy and resection specimen diagnoses. These findings indicate that diagnosis based on preoperative biopsy specimen should be interpreted with caution, and further investigation is required to enhance the accuracy of preoperative classification (35). Finally, the ability of the typical and atypical carcinoid classification alone to predict disease recurrence may be insufficient (34). Consequently, most patients undergo follow-up after surgical resection, while only up to 10% of patients will develop recurrence. For patients with typical carcinoids, recurrence occurs in up to 7%, whereas patients with atypical carcinoids have a risk of up to 35% of developing locoregional recurrence or distant metastases (36). Therefore, many patients undergo repeat imaging and prolonged follow-up of 10 years or even life-long, depending on the guidelines. This may unnecessarily increase radiation exposure and health care burden (37).

To address limitations in the current classification of pulmonary carcinoids, the integration of IHC markers as surrogates for underlying gene expression of *OTP* and *CD44*, together with Ki-67, may improve prognostic stratification and disease recurrence prediction. Multiple studies have demonstrated the additional prognostic value of Ki-67 in pulmonary carcinoids in recent years (5). In 2021, both the European Neuroendocrine Tumor Society (ENETS) and the North American Neuroendocrine Tumor Society recommended its inclusion as a complementary prognostic biomarker, and the incorporation of Ki-67 into the upcoming WHO classification is currently under discussion.

While the biological function of OTP in pulmonary carcinoids remains largely unknown, OTP has 1) proven to be a highly specific marker for pulmonary origin and 2) emerged as a robust prognostic biomarker capable of distinguishing more aggressive from less aggressive tumors (38, 39). Immunohistochemistry can be used to evaluate OTP in cytological and surgical specimens (40). Multiple studies have shown a strong association between loss/absence of nuclear OTP and a higher risk of recurrence or poor prognosis (29, 30, 38, 39, 41–43). Furthermore, high OTP expression is predominantly observed in the A1 and A2 subgroups, which are associated with a more favorable outcome, whereas loss of OTP expression is more frequently observed in the clinically aggressive B subgroup (12, 23, 25). CD44 is a cell-surface glycoprotein involved in cell adhesion, migration and cell-cell interactions. Similar to OTP, loss of CD44 protein expression is associated with an unfavorable clinical course, including an increased risk of distant metastasis and strongly associated with a high risk of recurrence (**Figure 5B**) (38, 39, 41, 42). In particular, the combination of nuclear OTP expression and membranous CD44 staining has shown strong predictive value for recurrence-free survival. This biomarker panel (OTP, CD44, and Ki-67) identifies patients with a low risk of distant recurrence who may not require prolonged follow-up (38). Moreover, this molecular approach appears to be less susceptible to interobserver variability than morphology-based classification alone.

Preoperative decision-making in pulmonary carcinoids can be challenging due to discordance between biopsy and resection diagnoses, particularly because it is currently impossible to distinguish typical from atypical carcinoids in small biopsies. As the extent of surgical resection (lobar *vs*. sublobar) is prognostically relevant in atypical carcinoid, optimal preoperative assessment is therefore crucial (36). Although biomarkers have been well validated in resection specimens, evidence in biopsies remains limited. Elevated Ki-67 (>3%) and loss of OTP expression are associated with worse outcomes, and combining OTP with CD44 and Ki-67 improves relapse prediction and interobserver agreement compared with the WHO 2021 classification (44)(van Weert et al., submitted). Biomarker expression in biopsy specimens must reliably reflect findings in the corresponding resection material for successful clinical implementation. Studies evaluating paired biopsy and resection samples generally demonstrate good concordance for Ki-67, OTP, and CD44 expression, although variability between studies highlights the need for standardized assessment and further prospective validation. Taken together, these findings suggest that biomarker-based risk stratification in biopsy material has the potential to meaningfully improve individualized preoperative decision-making in patients with pulmonary carcinoids, provided that its clinical utility is confirmed in future prospective studies.

Besides OTP, CD44 and Ki-67, other studies have demonstrated the prognostic value of telomerase reverse transcriptase (TERT) in pulmonary carcinoids. Werr et al. showed that TERT expression is low in patient with an indolent clinical course and elevated in patients with an unfavorable outcome, outperforming histologic classification and tumor stage as prognostic variables (45). This observation was further supported by Sexton-Oates et al., who reported an association between high TERT expression and worse prognosis in pulmonary carcinoids, also suggesting the possibility of TERT as molecular vulnerability in a subset of these tumors (46). Future studies should investigate the role of TERT across the molecular subgroups and evaluate its potential in guiding therapeutic decision-making (45).

Besides prognostic relevance, the molecular subgroups also entail diagnostic and therapeutic potential. Patients with A1 tumors are enriched for DLL3 and low in SSTR2A expression, whereas tumors belonging to the A2 or B subgroups show no DLL3 expression and high SSTR2A expression (25). *DLL3* in pulmonary carcinoids is relatively common, suggesting a potential for specific therapies (25, 47–49). DLL3 can be targeted with bispecific antibodies (50) and initial responses have been described in DLL3 positive carcinoid. This is complemented by new PET-CT tracers being established targeting DLL3 (51). Besides DLL3, significant differences have been observed for somatostatin receptor type 2a (SSTR2A) protein expression mainly in DLL3 negative subgroups. SSTR2a targeting also allows for tailored therapy and diagnostic imaging, for example through peptide receptor radionuclide therapy (52, 53) and 68Ga-DOTATOC PET-CT analysis. Hence, in the future a theragnostic approach targeting DLL3 or SSTR2a is likely in the management of (metastatic) LNET.

## Supra-carcinoids

### Molecular subgroups and potential biomarkers

In addition to the molecular subgroups A1, A2, and B, Alcala et al. identified an LNET entity with molecular and clinical characteristics of LCNEC (12, 13) so-called supra-carcinoid enriched. Utilizing state-of-the-art spatial omics and deep learning analysis additional insight has been provided of this specific molecular subgroup (46). Although supra-carcinoids exhibit more aggressiveness compared to other LNETs, these tumors show only a moderate increase in both Ki-67 and mitotic count compared with low- and intermediate-grade LNETs. This observation indicates that although supra-carcinoids are not necessarily defined as G3-NET, they may also fall within grade 1 or grade 2 NETs. These findings further suggest that tumors classified within the A1, A2, or B molecular subgroups can progress towards grade 3 tumors, highlighting the dynamic nature of molecular evolution within pulmonary carcinoids requiring further in depth assessment (9).

Compared with other cancer types, LNETs have relatively quiet genomes; however, each of the four molecular subgroups exhibit unique genomic features, including differences in copy number alterations and mutations (46). Notably, both the supra-carcinoids and A1 subgroups showed a high burden of small and structural variants (46). However, due to the more diverse mutational signatures, supra-carcinoids seem to undergo more mutational processes and have the largest number of upregulated genes, compared to A1, A2, and B. Small variant analysis revealed BRAF V600E mutation in a subset of supra carcinoids (2/14 cases). However, these findings should be validated in a larger cohort and to date it is unclear if supra-carcinoid is BRAF specific or may entail other driver mutation events considering the few cases evaluated.

### Clinical utility

Supra-carcinoids have an inferior prognosis compared to other LNETs, but due to a lack of clear biomarkers they currently cannot be identified in routine practice. Considering molecular vulnerability, TROP2, an oncogene encoded by the *TACSTD2* gene, was one of the core upregulated genes identified in supra-carcinoids. *TROP2* has been investigated as a target for antibody-drug conjugates (ADCs) in SCLC and may therefore represent a potential therapeutic option for supra-carcinoids in the future (46, 54). Immunotherapy and agents targeting telomerase activity, such as TERT inhibitors, could be potential molecular vulnerability for this molecular subgroup (46). However, the clinical validation of these approaches in supra-carcinoids still needs to be investigated.

## G3-NET

### Morphological recognition and biomarkers

The IASCL published an update to the classification of pulmonary neuroendocrine neoplasm, recognizing NET G3 as a distinct entity. This work provides a comprehensive overview of the available evidence, concluding that pulmonary NENs with morphologic features of a carcinoid tumor but increased proliferative activity (mitotic count >10/2 mm^2^ and/or higher Ki-67 >20%) are biologically more closely related to carcinoids than high-grade NECs and should therefore be classified and treated accordingly (55). This was based on a recent effort from the pathology committee of the international association for the study of lung cancer (IASLC)describing 27 tumors with an increased proliferation rate but conserved (e.g. well differentiated) morphology (11). These tumors had a mean mitotic index of 15 (ranging from 11-30) and Ki-67 index of 32% (ranging from 14-54) with wildtype TP53 and preserved pRb expression and decreased overall survival compared to atypical carcinoids. Previously such tumors had been recognized describing the relevance of retained pRb in this setting (8, 56).

The molecular overlap between atypical carcinoids and LCNEC with lower proliferation rates, which underlies the G3 concept, has been independently confirmed by two different studies. Simbolo et al. performed integrated transcriptomic and genomic profiling of atypical carcinoids and LCNECs separating three distinct molecular clusters irrespective of their histological classification. Cluster 1 was dominated by LCNECs with concurrent *TP53* and *RB1* inactivation and complete loss of pRb expression, consistent with SCLC-like biology. Cluster 3 was enriched for atypical carcinoids with frequent *MEN1* mutations and intact RB1/pRb, reflecting a carcinoid-like molecular profile (mean Ki-67 22%). Notably, Cluster 2 comprised a mixed population of both atypical carcinoids and LCNECs with intermediate molecular features, including co-occurring *TP53, MEN1*, and *RB1* alterations, and an intermediate prognosis with a mean Ki-67 of 37%. This intermediate cluster likely encompasses tumors that would today be recognized as G3-NET, and demonstrates that combined MEN1 and Rb1 immunohistochemistry can serve as a practical tool to trace malignant progression across the LNET-LCNEC continuum (57). Complementing these transcriptomic findings, Cros et al. characterized the genomic landscape of 11 high-grade pulmonary neuroendocrine tumors with carcinoid morphology, defined by Ki-67 >20% and/or >10 mitoses/2 mm², representing putative G3-NET. Genomic patterns were highly heterogeneous, ranging from quiet genomes to heavily rearranged tetraploid profiles, with most alterations targeting tumor suppressor genes rather than oncogenes. Recurrent losses of chromosomes 11, 3, and 13, overlapping with copy number patterns seen in carcinoid and aSCLC, provided further evidence of biological continuity between well-differentiated and high-grade carcinoid-morphology tumors (14, 58, 59). Importantly, spatial and temporal heterogeneity was demonstrated within individual cases, with paired samples from the same patient showing divergent genomic profiles, underscoring the dynamic and evolving nature of these tumors (6).

### Clinical utility

G3-NET may be recognized by the application of Ki-67 staining in the context of a bordering atypical carcinoid-LCNEC by currently WHO definition (**Figure 5C**). A preserved pRb and TP53 wildtype staining may further support its recognition. Previous studies have shown that these tumors have a poorer prognosis compared to other LNETs but better than the neuroendocrine carcinomas (11, 60, 61). This argues for a high frequent radiological follow-up after surgery and future studies should evaluate a role for adjuvant systemic treatment.

## Atypical SCLC

### Molecular subgroups and potential biomarkers

The recently described “atypical SCLC” adds another layer of heterogeneity to LNENs. SCLC is traditionally regarded as an entirely separate class of tumors compared to LNET (11). Although aSCLC displays the morphology of SCLC, these tumors demonstrate a close molecular relationship with carcinoids. Genomic alterations, including *MEN1*, *EIF1AX*, *ARID1A*, and *ATM*, frequently observed in pulmonary carcinoids, were also identified in aSCLC, with an enrichment of *ATM* mutations. In addition, several aSCLC described tumors show OTP immunohistochemical expression and a low tumor mutational burden (<2Mb) with high ASCL1 expression, which is more consistent with pulmonary carcinoids subgroup A1 than SCLC. Clinically, patients classified as aSCLC were never- or light smokers and lacked retinoblastoma1 (*RB1)* and *TP53* mutations or deletions, which is rarely the case in SCLC. Rekhtman et al. identified chromothripsis as an underlying genomic process of aSCLC. Chromothripsis is a process in which chromosomes undergo extensive fragmentation within a short timeframe, followed by incorrect repair, leading to complex rearrangements (11). This process was identified in 84% of the cases and was associated with *CCND1*, *CCND2*, *CDK4*, and *MDM2* extrachromosomal amplifications leading to *RB1* and *TP53* pathway dysregulation.

### Clinical utility

Identification of aSCLC should be considered in patients with a limited smoking history or unusually young age of SCLC onset. Whole genome sequencing may help identify chromothripsis and a low tumor mutation burden, while immunohistochemical evaluation of OTP may serve as additional marker (**Figure 5D**). Rekhtman et al. considered whether the currently investigated targeted therapies for SCLC, including DLL3 and seizure-related homolog protein 6 (SEZ6) directed therapies, could benefit patients with aSCLC. High protein expression levels of DLL3 and SEZ6 were observed, suggesting that these patients could benefit from therapies targeting these proteins. Additional therapeutic opportunities may include targets under clinical development targeting ecDNA-based driven oncogene amplifications, as well as inhibitors targeting CDK4 and MDM2 in tumors with amplifications and overexpression of these genes.

## LCNEC

### Molecular subgroups and potential biomarkers

The molecular profiles of LNENs differ substantially between carcinoids and LCNEC, with LCNEC displaying a higher somatic mutation burden (8.6 non-synonymous mutations per million base pairs) than carcinoids (17). Recent studies have identified two molecular subgroups of LCNEC. The first subgroup, also known as “type I LCNEC,” or “NSCLC-like” LCNEC, is characterized by frequent mutations in *KRAS*, *STK11*, and *KEAP1*, bi-allelic *TP53* mutations, and a lack of the SCLC hallmark *RB1* alterations. “Type II LCNEC,” also referred to as “SCLC-like”, are characterized by mutations in or loss of both *TP53* and *RB1* (17, 18). Driver alterations are more frequently observed in the NSCLC-like subgroup (18%) than in the SCLC-like subgroup (4%) (62).

Beyond the genomic SCLC-like and NSCLC-like dichotomy, transcriptional profiling has revealed that LCNEC harbors a rich landscape of molecular states driven by lineage-defining transcription factors. Building on SCLC subtyping in which ASCL1, NEUROD1, POU2F3, and YAP1 define four major subtypes, multiple recent studies have evaluated whether this NAPY framework translates to LCNEC. The NSCLC-like subgroup is characterized by a NE profile with high ASCL1 and DLL3 expression and low NOTCH expression. In contrast, SCLC-like LCNECs exhibited lower ASCL1, DLL3, CHGA, and SYP expression levels and higher NOTCH expression (1, 17). At the pan-cancer level, Wang et al. analyzed over 1,000 NECs from 31 tissue sites, identifying five intrinsic subtypes defined by ASCL1, NEUROD1, HNF4A, POU2F3, and YAP1 (63). In pulmonary LCNEC, subtype-Y was the most prevalent (38%), contrasting sharply with SCLC where subtype-A dominates (56%). The newly defined HNF4A-driven subtype-H constituted 13% of LCNECs and was characterized by preserved RB1 function, a gastrointestinal-like molecular signature and resistance to platinum/etoposide chemotherapy. The clinical biology of YAP1 in LCNEC was defined by Stewart et al., who demonstrated via IHC, genomics, and single-cell RNAseq that nuclear YAP1 expression, detectable in approximately 50% of LCNECs distinguishes two fundamentally distinct subsets (64). The YAP1-high subset is characterized by a mesenchymal and inflamed phenotype, retention of pRb, and enrichment for CDKN2A/B and SMARCA4 alterations. It also exhibits high PD-L1 expression and AXL upregulation, suggesting potential vulnerability to AXL-targeted therapies as well as immunotherapy. The YAP1-low subset exhibits an epithelial and immune-cold phenotype, is enriched for *TP53/RB1* co-mutations, and is characterized by high expression of ASCL1, NEUROD1, DLL3, and CD56, supporting the potential use of DLL3-targeted therapies. Hence, transcriptionally LCNEC remains very heterogeneous with different studies highlighting unique patterns newly identifying YAP1 and HNF4A as transcriptional factors of interest.

The pathological feasibility of transcription factor subtyping in routine practice was established by Heijboer et al. and Hung et al. in two complementary IHC studies (65, 66) while others have reported smaller (67–72) cohorts. Heijboer et al. applied the NAPY panel to 133 centrally reviewed resected LCNECs, identifying five expression-based subgroups: NEUROD1^high^-ASCL1^high^ (10%), ASCL1^high^ (22%), POU2F3^high^ (5%), YAP1^high^ (11%), and NAPY^low^ (51%). Neuroendocrine subgroups were DLL3^high^ and pRb-deficient, while YAP1^high^ was enriched for pRb retention and showed an immune-inflamed profile on spatial transcriptomics. Critically, over half of LCNECs lacked any dominant NAPY marker, underscoring the greater transcriptional heterogeneity of LCNEC compared with SCLC. Hung et al. applied a five-marker panel including HNF4A to 117 consecutive LCNEC and high-grade NEC cases, finding ASCL1 dominant in 62%, followed by YAP1 (17%), NEUROD1 (8%), POU2F3 (6%), and HNF4A (2%). Importantly, YAP1 and HNF4A H-scores each correlated with large-cell morphology, while NEUROD1 dominance was more frequent in combined/intermediate morphology cases bridging LCNEC and SCLC. HNF4A was further characterized by Asayama et al., who identified HNF4A^high^ cases in 15% of resected pulmonary NECs (73). These tumors were almost exclusively ASCL1 or NEUROD1 subtype, carried a gastrointestinal signature with CDX2 co-expression, and showed significantly worse prognosis in LCNEC. NSCLC like NFE2L2/KEAP1 mutations were characteristic of HNF4A-positive LCNECs. Based on the available cohorts, the previously reported POU2F3 (SCLC) subtype is rare in pure LCNEC and associated with overlapping cytomorphological features resembling SCLC (e.g. intermediate- SCLC) loss of pRb and cMYC overexpression (67, 69).

The emerging picture from these studies reframes LCNEC as a transcriptionally heterogeneous disease in which subtypes, defined by ASCL1-NEUROD1 with overlapping HNF4A expression *vs.* YAP1 with a smaller role for POU2F3.

### Clinical utility

No standard treatment exists for LCNEC, and chemotherapy including immunotherapy has limited efficacy (18, 74). The subdivision into SCLC-like and NSCLC-like groups has not yet led to improved therapeutic outcomes although different chemotherapy regimens maybe applicable showing different response and progression free survival (15, 75, 76). Importantly, molecular profiling has identified actionable oncogenic alterations and this is enriched in NSCLC-like LCNEC. Furthermore, targeted treatment may improve overall survival compared to chemotherapy regimens mainly in NSCLC-like LCNEC (62, 77).

Based on recent studies, several new druggable targets of have emerged. Nassar et al, evaluated possible actionable targets in genomically defined LCNEC subtypes. The NSCLC-like LCNECs displayed high RNA expression of *FGL-1* and *SPINK1*, suggesting a potential benefit from targeting the LAG-3 or SPINK1 pathways (18). Besides, *DLL3* was defined as a therapeutic target for both genomically NSCLC-like and SCLC-like LCNEC. Previous studies have shown expression of DLL3 in 74-80% in (stage IV) LCNECs through immunohistochemistry (78, 79). However, first-generation DLL3-targeted ADCs demonstrated limited clinical benefit in high-grade NECs (80). Ongoing clinical trials are now evaluating alternative DLL3-targeting strategies, including T-cell engagers, showing promising efficacy in LCNEC (81–83). SEZ6, a transmembrane protein involved in neuronal development, is another potential target for ADC therapy. High SEZ6 expression has been identified in high-grade NECs, including LCNEC, and ongoing studies are exploring SEZ6 as a novel therapeutic target for NECs (84). In addition, B7-H3 (CD276), an immune checkpoint molecule, is frequently overexpressed in many cancer types, including SCLC (85, 86). Since the SCLC-like subgroup is characterized by loss of *TP53* and *RB1*, sharing key biological features with SCLC, this suggest that B7-H3 may also be a promising therapeutic target in this LCNEC subgroup utilizing antibody drug conjugates (86).

Summarizing molecular vulnerabilities and transcriptional states described in LCNEC; ASCL1^high^ and NEUROD1^high^ tumors are DLL3-rich and more often pRb-deficient, potentially favoring DLL3/SEZ6-targeted and platinum/etoposide approaches. The YAP1^high^ tumors are immune inflamed and frequently pRb-retained, potentially favoring immunotherapy by PD1/PD-L1 inhibition and LAG3 inhibition, while POU2F3^high^ are rare in pure LCNEC and generally associated with intermediate cell type. HNF4A is associated with ASCL1-NEUROD1-DLL3 expression and a NSCLC genomic subtype potentially more resistant to chemotherapy treatment (69). Prospective studies evaluating the transcriptional states in association with treatment outcome are urgently needed to assess its value in routine clinical decision-making for LCNEC.

## Discussion

LNENs, particularly carcinoids and LCNEC, represent a group of heterogeneous tumors for which the current classification and patient management are mostly based on morphology. However, morphology alone is insufficient for defining the underlying biological and prognostic variability and therapeutic vulnerabilities of these tumors. This review was written to describe the current knowledge on molecular subgroups, their distinct molecular profiles, and potential molecular vulnerabilities in pulmonary carcinoids and LCNEC. To improve patient management, there is an urgent need for personalized therapeutic approaches, identifying patients with a higher risk of disease recurrence, and avoid unnecessary prolonged follow-up in patients.

For pulmonary carcinoids, the ENETS recommends a long-term follow-up of 15 years, and the European Society for Medical Oncology (ESMO) recommends a life-long follow-up (36). Ideally, an approach is established that excludes patients at low risk of recurrence from long-term follow-up (87) and improved recognition of potential recurrences. Pulmonary carcinoids with a loss of CD44 or OTP or an elevated Ki-67 have a higher risk of developing a recurrence, which can be helpful in patient surveillance and adjusting follow-up times for specific patient groups. Ongoing research on these prognostic markers will improve their specificity.

Integrated multi-omics studies have provided crucial insights, revealing that pulmonary carcinoids are not a uniform entity but consist of distinct molecular subgroups (A1, A2, and B) with specific clinical, genomic, and transcriptional features. Although recurrent alterations in chromatin-remodeling genes such as *MEN1*, *ARID1A*, and subunits of the SWI/SNF complex characterize many carcinoids, these alterations lack direct therapeutic implications. Importantly, subgroup-specific markers, such as OTP, ASCL1, and HNF1A, can be detected at the protein level, suggesting that IHC panels may allow the implementation of molecular subclassification in routine practice. More recent studies have suggested the existence of additional subgroups; grade 3 NET, supra-carcinoids and aSCLC, further highlighting the biological coherence between carcinoids and high-grade NENs (6, 14, 46, 57). The IASLC proposes harmonizing the classification of pulmonary carcinoids with that of GEP NETs by introducing the terminology carcinoid/NET G1 and carcinoid/NET G2 for typical and atypical carcinoids respectively (55).

In LCNEC, molecular profiling has uncovered two major subgroups with distinct genomic drivers: SCLC-like subgroup characterized by concurrent *TP53* and *RB1* alterations and an NSCLC-like subgroup with *STK11*, *KEAP1*, and *KRAS* mutations typical of NSCLC. These molecular differences are potentially clinically relevant for choice of treatment. The NSCLC-like subtype more often has actional driver mutations relevant for clinical screening. Additionally, the expression of transcription factors, such as NEUROD1-ASCL1-HNF4A, YAP1 and POU2F3 can further distinguish LCNEC subgroups, potentially providing additional insight into their behavior and treatment response. Potential therapeutic targets, such as DLL3, SEZ6, and B7H3, are currently under investigation for ADCs/t-cell engager directed approaches.

Future research should focus on the integration of molecular profiling into routine diagnostics. Larger cohort studies combining multi-omics layers will be essential to identify prognostic biomarkers and novel molecular vulnerabilities and refine the subgroups. Prospective studies are needed to evaluate potential molecular vulnerabilities defined by molecular subgroups to improve clinical outcomes. Finally, molecular-driven management may enable personalized treatment approaches, reduce unnecessary surveillance, and improve prognosis for patients with pulmonary carcinoids and LCNEC.
